# Design and Error Analysis of an Optical Measurement System for the Wavefront of Large-Aperture Segmented Mirror

**DOI:** 10.3390/s26051450

**Published:** 2026-02-26

**Authors:** Yukun He, Hongbo Zhao, Lanxin Peng, Xiaodong Sui, Changzheng Chen, Yueyang Peng

**Affiliations:** 1 Changchun Institute of Optics, Fine Mechanics and Physics, Chinese Academy of Sciences, Changchun 130033, China; heyukun18@mails.ucas.ac.cn (Y.H.);; 2 University of Chinese Academy of Sciences, Beijing 100049, China; 3 School of Instrument Seicence and Opto-Electronics Engineering, Hefei University of Technology, Hefei 230009, China

**Keywords:** segmented mirror, wavefront measurement, optical measurement, wavefront fitting, optical remote sensor

## Abstract

To better meet the wavefront measurement requirements for large-aperture segmented mirrors after in-orbit deployment, this paper designs a measurement system based on an optical camera and targets. This system utilizes photogrammetry principles to measure target positions, fit the wavefront of the segmented mirror, and form a closed-loop control with the calibration mechanism. Based on the wavefront measurement range and accuracy requirements during the coarse calibration phase of the segmented mirror, the optical system was first designed. The measurement camera features a 16° × 12° rectangular field of view with a 100 mm focal length, achieving near-diffraction-limited imaging quality. The structural fundamental frequency of the measurement camera exceeds 400 Hz. Under a 4 °C temperature rise environment, the surface error of the optical lens remains better than 1/80λ. Based on error theory, a quantitative analysis of error sources and their impact on target position measurement accuracy was conducted, yielding theoretical measurement errors of ±0.0853 mm in the Z-direction and ±0.1525 mm in the X-direction. Through focal length calibration and imaging tests of the prototype, the measurement camera achieved a modulation transfer function greater than 0.11 with excellent imaging quality. With a focal length of 101.356 mm and a measurement range exceeding ±4 mm, it meets design requirements. Finite element simulation and Monte Carlo methods analyzed wavefront fitting accuracy under different operating conditions, yielding peak-to-valley values of 0.397 mm and root mean square values of 0.073 mm. The wavefront measurement system designed in this paper meets the structural rigidity and temperature adaptability requirements for in-orbit measurement systems. The prototype’s field of view satisfies the wavefront measurement range requirements, the camera’s focal length meets design specifications with good imaging quality, and the wavefront measurement deviation meets the accuracy requirements for the coarse calibration phase. Compared to current wavefront measurement systems, the proposed system significantly expands the measurement range, offering a novel wavefront measurement method for coarse calibration of tiled mirrors.

## 1. Introduction

With the rapid advancement of aerospace optical remote sensing technology, space-based optical cameras are increasingly applied in military, agricultural, surveying, and meteorological forecasting fields. As a critical component of optical cameras, the aperture of the mirror directly determines the core performance metrics of the camera. A large-aperture mirror effectively enhances the optical system’s resolution, thereby improving the camera’s target recognition capability during Earth observation. The field of view (FOV) of an optical camera is similarly dependent on the mirror aperture. Wide-field cameras can capture a larger observation area in a single imaging mission [1], thereby increasing the camera’s Earth observation efficiency and effectively reducing the revisit cycle of space cameras. Large-aperture mirrors provide optical cameras with superior light transmission capabilities, improving the camera’s transfer function and signal-to-noise ratio, which significantly enhances imaging quality. However, due to limitations imposed by rocket fairing diameters, large-aperture monolithic mirrors are typically restricted to 2 to 3.5 m in diameter [2].

To increase the aperture of optical cameras, segmented mirrors and their wavefront measurement technology have garnered significant attention [3,4]. Segmented mirrors typically consist of at least two sub-mirrors. The backs of these sub-mirrors are mounted on a folding mechanism via six-axis adjustment actuators. During launch, the folding mechanism retracts to reduce volume. After orbital deployment, the mechanism unfolds, assembling the sub-mirrors into a single mirror surface. Each sub-mirror requires positional calibration via the six-axis adjustment mechanism [5]. A wavefront sensor continuously measures the wavefront error of the assembled mirror surface, feeding this data back to the adjustment mechanism until the error meets optical imaging requirements. Gao Xu [6] proposed a streak reflection-based detection technique for large-aperture reflectors. For mirrors around 1.5 m in diameter, this method enhances detection accuracy by a factor of 12 within a 0.2392 mm dynamic range. Acton D S et al. [7,8] proposed a wavefront measurement method combining phase recovery with image defocusing. With a measurement range of approximately 1 mm, it integrates coarse and fine co-phasing through iterative calculations. The root mean square (RMS) error of wavefront measurement is better than 10 nm, validated through actual testing on the JWST space telescope. Additionally, the long-wavelength channel of the Near-Infrared Camera incorporates a prism equipped with Dispersed Fringe Sensing (DFS). During coarse phase alignment, the measurement range spans 500 μm [9]. The Extremely Large Telescope (ELT) employs inductive edge sensors [10]. Each inductive edge sensor achieves a measurement accuracy of 1 nm, with a range of 400 μm and a temperature drift of 1.32 nm/°C [11]. Dispersed Fringe Sensing (DFS) utilizes Fraunhofer double-aperture diffraction and dispersion of polychromatic light, enabling a large measurement range (approximately ±100 μm). Its drawback is lower detection sensitivity (accuracy around 100 nm) [12,13]. Wavefront measurements in the primary mirror of the Keck Observatory are performed by the Phasing Camera System, which features four operational modes. These modes work in concert to achieve measurement accuracy better than 50 nm (RMS) within a ±30 μm range [14]. The Thirty Meter Telescope improved upon Keck’s Phasing Camera System. Based on the Shack–Hartmann principle, the Thirty Meter Telescope’s wavefront measurement system achieves a measurement accuracy better than 30 nm (RMS) within a ±30 μm range [15]. Typically, a larger measurement range results in lower measurement accuracy. Modern large-aperture segment telescopes [7,16] invariably adopt a hierarchical strategy: utilizing large-range technology for millimeter-to-micrometer-level capture, followed by intermediate-range technology for transition, and finally small-range technology for nanometer- or sub-nanometer-level precise positioning.

Factors such as mechanical friction, clearances, and localized contact collisions contribute to deployment errors in folding mechanisms [17], and in microgravity conditions, deployed folding mechanisms undergo deformation [18], ultimately causing wavefront errors on the assembled mirror surface to exceed the measurement range of wavefront or edge sensors. Therefore, coarse calibration must first bring the wavefront error of the assembled mirror surface within the sensor’s measurement range. Subsequently, precision calibration employs wavefront detection and active optical correction techniques to control the optical system’s wavefront error within optical tolerances. This ultimately enables the surface accuracy of the assembled primary mirror to approach that of a monolithic mirror. Consequently, designing a wavefront measurement system suitable for the coarse calibration stage capable of measuring the wavefront of the joined mirror over a large range and forming a closed-loop system with the adjustment mechanism to calibrate the wavefront aberration within the sensor’s measurement range is critical to ensuring successful wavefront alignment of the joined mirror. This paper presents an optical measurement system for assessing wavefront errors in segmented mirrors. Utilizing an optical camera and targets, the system employs photogrammetry principles to calculate and fit the wavefront of the segmented mirror by measuring target positions. The main contributions of this paper are as follows:

1. This paper investigates wavefront measurement methods for tilting mirrors based on photogrammetric principles, designing an optical imaging-based wavefront measurement system for in-orbit deployed tilting mirrors;
2. Considering the in-orbit operational environment of the wavefront measurement system, this paper conducts optical design, structural design and simulation analysis, thermal control design, and electronics design for the optical measurement camera, followed by camera integration testing and calibration; To address the in-orbit illumination environment, an active target is designed to ensure normal camera imaging under low-illumination conditions;
3. Considering the distribution of the measurement camera and target, along with the characteristics of the rectangular mirror, a coordinate transformation and wavefront fitting algorithm are proposed. The wavefront measurement accuracy of the system is analyzed through finite element simulation and Monte Carlo analysis.

This paper is organized as follows: Section 2 briefly introduces the measurement principle and the installation methods for the target and measurement camera. Section 3 details the design of the measurement system, including the camera design, target design, and fitting algorithm design. Section 4 quantitatively analyzes the impact of various errors on measurement accuracy through error theory. Section 5 conducts imaging tests of the measurement camera and simulation analysis of wavefront fitting deviations. Section 6 and Section 7 present the discussion and conclusions.

## 2. Measurement Principle

**Figure 1:**
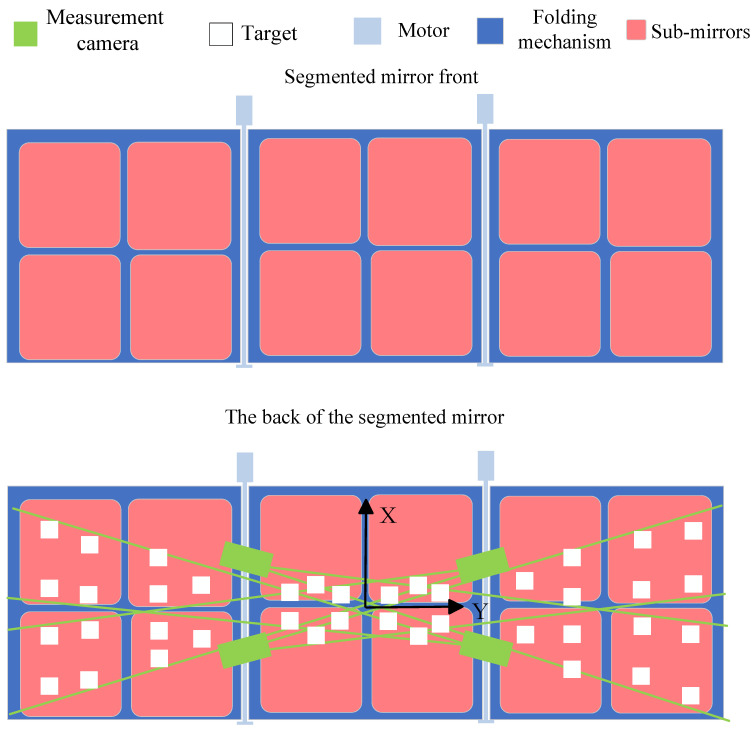
Overall layout of the optical measurement system.

The segmented mirror studied in this paper is shown in Figure 1. The sub-mirrors are mounted on a 6 m × 2 m rectangular panel composed of three support plates, each holding four sub-mirrors. In the stowed configuration, the side support plates fold backward around the X-axis to lie close to the satellite body. In the deployed configuration, the side support plates unfold, and the 12 sub-mirrors assemble into a single primary mirror.

**Figure 2:**
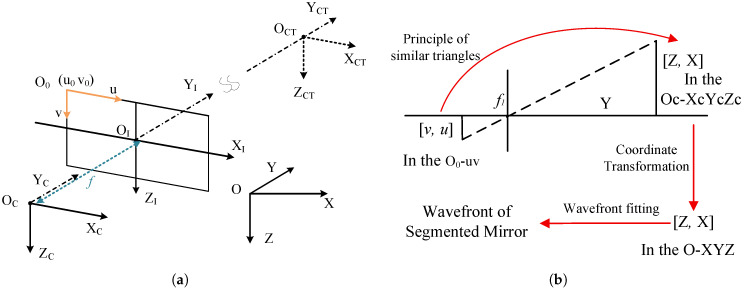
Principle: (**a**) coordinate definition. (**b**) Principles of triangulation and steps of wavefront fitting.

Place a target and camera on the back of the sub-mirror. The camera captures an image of the target. From the target image, extract the target center and calculate its position within the image coordinate system. Using the camera’s intrinsic parameters (such as focal length and pixel size), compute the target’s position (X, Z axes). Finally, fit the target position to the wavefront equation. The coordinate systems relevant to target position measurement are illustrated in Figure 2a. The camera’s line of sight direction $O-Y_{l}$ corresponds to $O_{c}Y_{c}$ in the figure below. Here, $O_{c}-X_{c}Y_{c}Z_{c}$ represents the camera coordinate system, while $O_{I}-Y_{I}Z_{I}$ denotes the image plane coordinate system. $O_{c}Y_{c}$ and $O_{c}Z_{c}$ are parallel to $O_{I}-Y_{I}$ and $O_{I}Z_{I}$, respectively, and $O_{c}X_{c}$ is perpendicular to the image plane coordinate system, passing through its origin. $O_{0}-uv$ represents the image coordinate system. The $O_{I}-Y_{I}Z_{I}$ plane coincides with the $O_{0}-uv$ plane, with their origins separated by $\left(u_{0},v_{0}\right)$. $O_{CT}-Y_{CT}Z_{CT}Z_{CT}$ constitutes the target coordinate system, where $O_{CT}$ denotes the center point of the target. O−XYZ is the reference coordinate system of the folding mechanism. In the camera coordinate system, the measurement principle for the target’s position (X, Z axes) is illustrated as Figure 2b.

By the principle of similar triangles, we can obtain $Z=\frac{vY}{f_{1}}dy$, and similarly, we can derive $X=\frac{uY}{f_{1}}dx$. (Z,X) represents the target’s coordinates in the camera coordinate system. (u,v) denotes the position coordinates of the target’s center point in the image coordinate system. dy and dx denote the size of the pixel and $f_{1}$ denotes the focal length. After obtaining the target position (Z,X) in the camera coordinate system, coordinate transformation converts (Z,X) to the reference coordinate system of the folding mechanism O−XYZ. Wavefront fitting is ultimately performed in the O−XYZ coordinate system.

## 3. Measurement System Design

### 3.1. Overall Design

**Figure 3:**
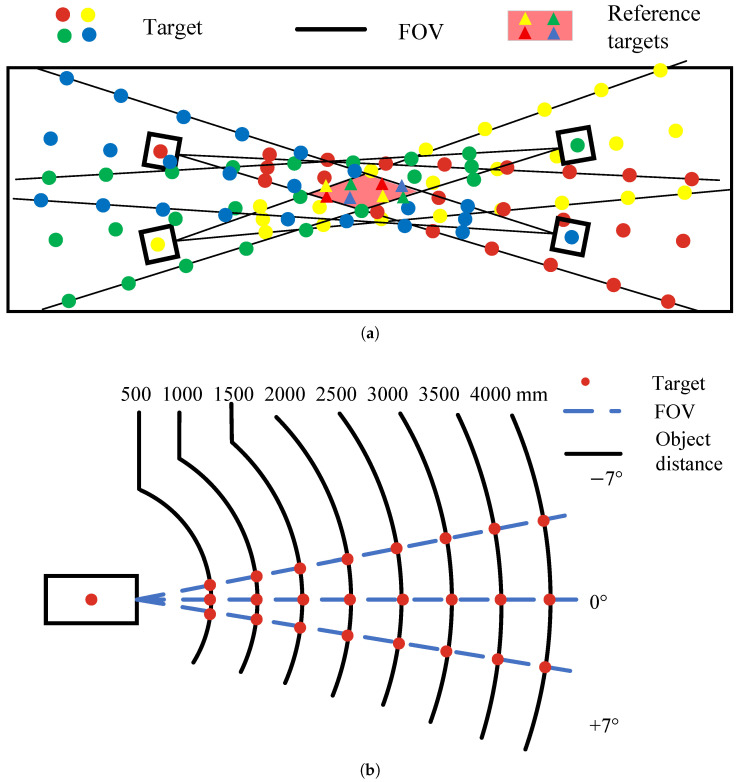
Overall design of the measurement plan: (**a**) camera and target distribution. (**b**) Single-camera target distribution.

The optical measurement system of large-aperture segmented mirrors comprises multiple active targets and four optical measurement cameras. The installation configuration of cameras and targets is illustrated in Figure 3a. For a single camera, the target distribution is shown in Figure 3b. To maximize the utilization of the camera’s FOV and increase coverage area, targets are distributed across the camera’s ±7° FOV and central FOV, uniformly spaced along the object distance. As shown in the layout diagram, the four cameras collectively image 96 targets, with each camera capable of measuring the positions of 24 targets. By simultaneously imaging eight reference targets with all four cameras, these targets serve as the measurement baseline. This enables the unification of the four camera coordinate systems, facilitating surface fitting of the segmented mirror.

The measurement range of the wavefront during the coarse calibration stage depends on the unfolding error and maximum deformation of the folding mechanism. Considering these factors, based on finite element analysis, considering the unfolding error of the folding mechanism and thermal deformation, the wavefront PV values of the assembled mirror under different temperature conditions are shown in Table 1. Under the combined effects of mechanical expansion and thermal deformation, the maximum PV value of the wavefront for the segmented mirror reached 3.785 mm. To ensure an adequate measurement margin, the measurement range of the wavefront measurement system was set to ±4 mm. The measurement accuracy is determined by optical tolerances and the measurement range of the wavefront sensor. If the deviation between the measured wavefront and the actual wavefront is too large after the coarse calibration stage, the precision calibration stage cannot proceed. Therefore, during the coarse calibration stage, the wavefront must be calibrated within the measurement range of the wavefront sensor. According to reference [19] SFD wavefront sensor has a measurement range of 500 μm (PV). The optical tolerance of the tilted mirror studied in this paper is λ/5 = 0.126 μm (PV). Therefore, the measurement accuracy should be 499.874 μm (PV). For optical surfaces, PV is typically 3 to 5 times RMS, though this ratio is not a fixed constant [20]. This paper sets the PV-to-RMS ratio at 4.5 times. In summary, the key specifications of the optical measurement system are shown in Table 2.

### 3.2. Optical Design

Measurement cameras typically employ image-side telecentric optical systems. Even with slight shifts in the camera’s imaging plane position, the size and location of the target image remain unchanged, significantly reducing imaging distortion caused by focal length variations. Additionally, telecentric optical systems on the image side minimize image distortion resulting from lens curvature and field-of-view differences, ensuring measurement accuracy [21].

**Figure 4:**
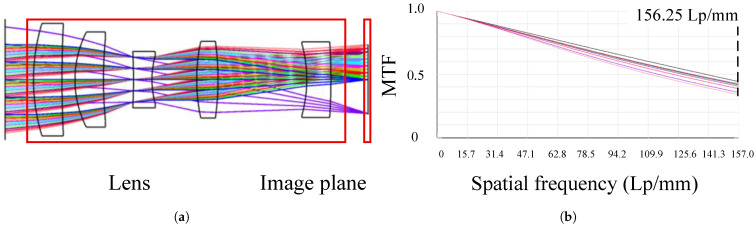
Result of optical design: (**a**) optical path diagram of camera. (**b**) For MTF curves of the camera, during optical design, the MTF at the Nyquist frequency should be evaluated. The CMOS detector pixel size of the camera is 3.2 μm, so the Nyquist frequency is $\frac{1}{2\times 3.2\times 10^{-3}}=156.25\;\mathrm{Lp}/\mathrm{mm}$.

To achieve a large FOV, a transmissive system is employed. To minimize interference from sunlight, the operational wavelength band is set to the red band (center wavelength 700 nm). The optical path, as shown in Figure 4a, comprises four lenses and a focal plane. The rear surfaces of Lenses 2 and 3 feature even-order aspheric surfaces. The camera’s modulation transfer function (MTF) curves are shown in Figure 4b, where different colors and line styles represent MTF curves for the meridian and tangential directions across various FOVs. With maximum distortion better than 0.20%. Optical design analysis indicates that the optical system’s transfer function exceeds 0.3. Based on engineering experience [22], this optical system delivers good imaging quality.

### 3.3. Camera Structural Design and Finite Element Analysis

**Figure 5:**
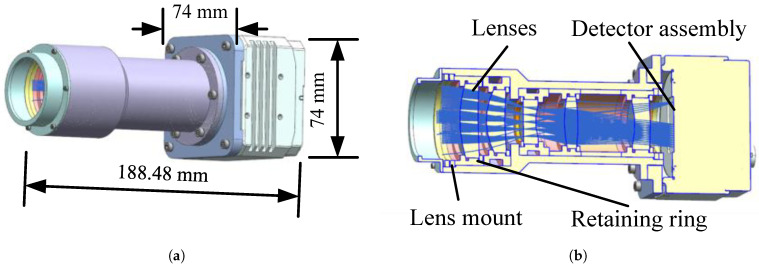
Optomechanical structure: (**a**) exterior view; the camera’s length, width, and height are 188.48 mm, 74 mm, and 74 mm, respectively. (**b**) Sectional view.

Each lens can be directly secured within the lens mount via a threaded retaining ring. A single lens assembly consists of a lens mount and lens retaining ring. The lens retaining ring and lens mount are connected by threads, with the lens fixed to the mount by spin-coated silicone rubber. As shown in Figure 5.

During assembly of individual lens components, optical and mechanical properties are calibrated using instruments such as laser interferometers and autocollimators. After applying black anti-glare paint around the lens periphery, silicone rubber is coated onto the outer wall of the lens mount. Once the lens is securely positioned, the retaining ring is pressed into place and adhesive is applied. During the centering turning stage, an optical centering instrument precisely aligns the lens optical axis with the lathe spindle axis. The outer circumference of the lens mount is machined to ensure post-assembly eccentricity and tilt meet tolerance requirements, while confirming compliance with outer diameter and perpendicularity specifications. After turning the rear end of the lens mount, adjust the spacing to meet lens spacing tolerances. During detector assembly, install the connecting flange, grind the mounting flange to adjust tilt, use mounting screws to adjust eccentricity, and machine the mounting flange to adjust spacing. Titanium alloy has a density of approximately $4.4\;{g/\mathrm{cm}}^{3}$. Titanium alloy is selected for the lens support frame, barrel, mount, and clamping ring. Material parameters are shown in Table 3. The total weight is shown in Table 4, with a total mass of 1.235 kg.

Based on the structural model’s different structural types, perform relevant element partitioning to establish an accurate finite element model. Carefully inspect and eliminate elements exhibiting disproportionate scaling or severe distortion, either individually or in relation to one another. Simultaneously, establish reasonable connections based on the interfaces between components within the structural model and between the components and the platform. Perform engineering analysis of the entire assembly using finite element analysis software. Since the entire assembly connects to the installation plate via three mounting bosses on the main support plate, the constraint conditions fully constrain all six degrees of freedom at each interface node. When simulating screw connection assembly relationships, place a node at the center of the threaded hole and rigidly connect the nodes on the edges of the mating surface threaded holes. A contact model was established between the primary mirror and the flexible pressure block. For the secondary mirror mount and secondary mirror bonding assembly simulation, the bonding surfaces were connected using TIE to prevent relative motion between the two contact surfaces.

The camera’s optical-mechanical structure system must satisfy imaging requirements for the optical reflector surface under various ground manufacturing and in-orbit operational conditions. This ensures overall assembly and in-orbit imaging meet specification requirements, meaning the displacement and surface profile changes in all camera optical components must remain within design tolerances under the conditions described below.

**Figure 6:**
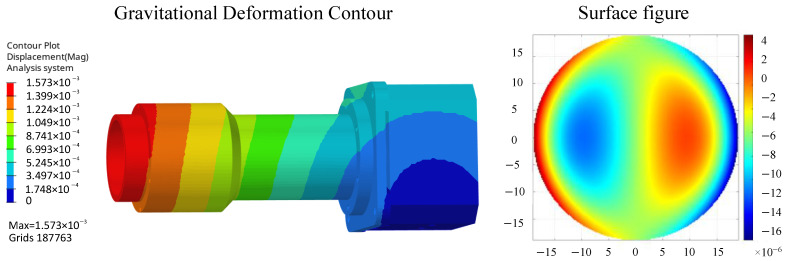
Gravity conditions (mm).

The camera’s static analysis primarily addresses deformations caused by altered gravity conditions and temperature variations. The gravity scenario refers to the condition where microgravity induces deformation of the mirror surfaces. The deformation analysis model under gravity is shown in Figure 6, with the optomechanical analysis integration results presented in Table 5.

**Figure 7:**
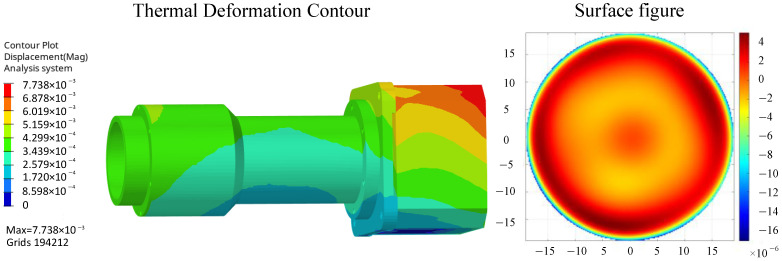
24 °C temperature condition, reference temperature 20 °C (mm).

Temperature rise conditions refer to scenarios where the mirror deforms within the camera’s operating temperature range of 20 ± 4 °C. The 4 °C temperature rise deformation analysis model is shown in Figure 7, with analysis results presented in Table 5. with the optomechanical analysis integration results presented in Table 5. Excessive surface profile errors in optical mirrors can induce wavefront aberrations in optical systems, degrading the imaging quality of measurement cameras. According to optical tolerance requirements for surface profile accuracy, the RMS surface profile error of measurement camera lenses is less than 1/80λ (8.75 nm), resulting in minimal impact on imaging quality.

Modal analysis provides the basis for analyzing the vibration characteristics of structural systems and optimizing the design of structural dynamic properties. Modal analysis must consider at least 88% of the effective mass to ensure its accuracy. The results of finite element dynamic analysis indicate that the first three resonance frequencies of the entire system are relatively high (more than 400 Hz). This demonstrates that the mirror assembly meets the structural dynamic stiffness requirements and possesses the capability to resist low-frequency vibrations.

**Figure 8:**
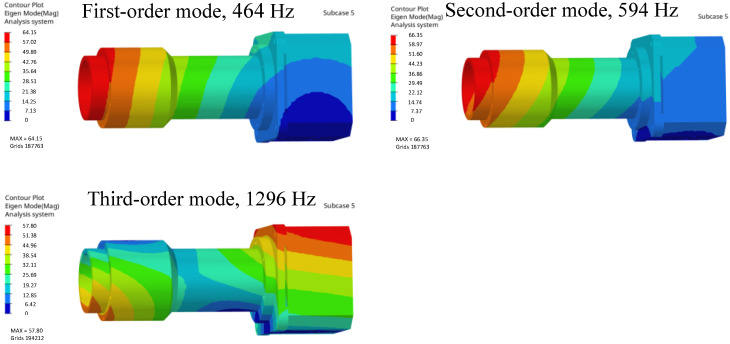
Schematic of modal results.

Modal analysis of the opto-mechanical structure serves as a key indicator for characterizing its dynamic stiffness. By obtaining the first three modes through modal analysis, the camera’s dynamic stiffness can be evaluated. Based on the analysis, the first three modes of the camera are shown in Figure 8.

### 3.4. Camera Imaging Electronics and Controllers

The imaging electronics system of the measurement camera employs a highly integrated CMOS area-array photodetector, model GMAX3265. It features an on-chip SPI controller, timing controller, and analog-to-digital conversion quantization functionality with a maximum quantization resolution of 12 bits, as shown in the Table 6.

**Figure 9:**
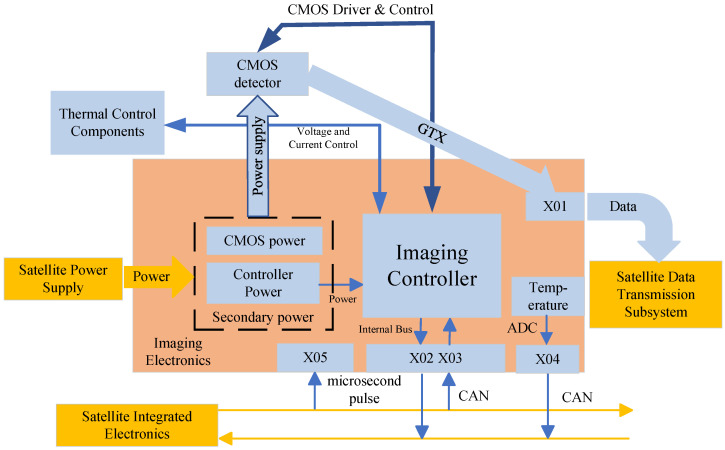
Camera electronics system.

The camera electronics system comprises a CMOS imaging unit, camera controller, base platform, secondary power supply, and other components, as shown in Figure 9. The CMOS focal plane imaging circuit is located on the focal plane assembly. The camera controller, secondary power supply, and base platform are integrated as a standalone unit mounted on the installation plane, connected to the camera via cables. The CMOS focal plane imaging unit within the camera electronics system includes one CMOS sensor and corresponding processing circuitry. It transmits raw image data to the satellite’s data transmission subsystem via a high-speed Gbit interface. The camera controller manages external communication with the satellite’s integrated electronics system and telemetry/command system, while internally communicating with the CMOS imaging unit and implementing active thermal control functions. The secondary power supply provides power to both the camera controller and the CMOS imaging unit.

### 3.5. Thermal Control Design

The primary purpose of camera thermal control is to ensure structural precision under space temperature conditions, thereby guaranteeing imaging quality. Key design features of the camera thermal control system include the following:

1. Thermal isolation between the camera and mounting platform;
2. The camera’s light-transmitting aperture faces a cold black space long-term, maintaining a stable thermal environment. The camera employs a combination of active and passive thermal control to maintain temperature levels, temperature gradients, and thermal stability;
3. Clear interface configuration between imaging electronics and camera lens components; imaging electronics utilize independent cooling with corresponding thermal compensation;
4. Internal thermal conduction design within imaging electronics to transfer heat from CMOS and high-power components to the imaging electronics heat sink.

Thermal control solutions comprise active and passive thermal management. Passive thermal control design includes the following:

1. Thermal isolation between the camera and mounting platform. Thermal pads made of 7 mm-thick polyimide are used between the camera mounting bracket and the platform;
2. Thermal insulation between the camera and the external environment. The camera’s outer surface is covered with multi-layer thermal insulation components to insulate it from the external environment. The multi-layer components consist of 20 units, each with a film-mesh structure. The multi-layer film is made of silver-plated F46 film;
3. Heat dissipation design for imaging electronics components. Based on the folding mechanism coordinate system and camera mounting orientation, where +Y and −Y represent the camera’s optical axis direction, the ±X surfaces of the imaging electronics components can serve as heat dissipation surfaces;
4. Internal thermal conduction design for the focal plane electronics box Thermal management measures are implemented for the CMOS and high-power components, directing heat transfer to the ±X surfaces of the electronics components.

Active thermal control design includes the following:

1. Calculation of active thermal control power consumption for the camera lens section. With a camera aperture diameter of 50 mm, an equivalent emissivity of 0.9, and an equivalent temperature of −10 °C, the long-term active thermal control power requirement for the camera lens section is approximately 0.3 W, calculated using the Stefan-Boltzmann law;
2. Thermal resistance calculation between the camera mount and installation platform. The total contact area between the camera mount and the folding mechanism platform is approximately 714 mm^2^. With the camera mount and thermal pad installed normally, using a heat transfer coefficient of 400 W/m^2^/°C, the thermal resistance R1 between the camera mount and thermal pad is calculated as 3.50 °C/W. Similarly, the thermal resistance R2 between the installation platform and thermal pad is also 3.50 °C/W. The total area of the polyimide thermal pad is approximately 714 mm^2^, with a thickness of 0.7 mm. Using a polyimide thermal conductivity of 0.3 W/m/°C, the thermal resistance R3 of the polyimide thermal pad is calculated as 3.26 °C/W. In summary, the total thermal resistance R between the camera mount and folding mechanism is R = R1 + R2 + R3 = 10.26 °C/W. When the mounting interface temperature is −10 °C, the heat leakage from the camera mount is approximately 2.9 W;
3. The long-term power consumption requirement for the camera’s active thermal control is 0.3 + 2.9 W;
4. The camera’s active thermal control is designed with three heating zones. Each heating zone is controlled by one main and one backup heating circuit. The heaters are cold-backed up, and the temperature sensing elements are hot-backed up. The active temperature control element uses an MF501 thermistor (Murata Manufacturing Co., Ltd., Nagaokakyo, Kyoto, Japan), with temperature control accuracy better than 0.2 °C and interchangeability accuracy better than 0.5 °C. The camera heating zone design is shown in Table 7. The design power for each section is to be determined, with a total power of approximately 3.2 W;

### 3.6. Design of Target

**Figure 10:**
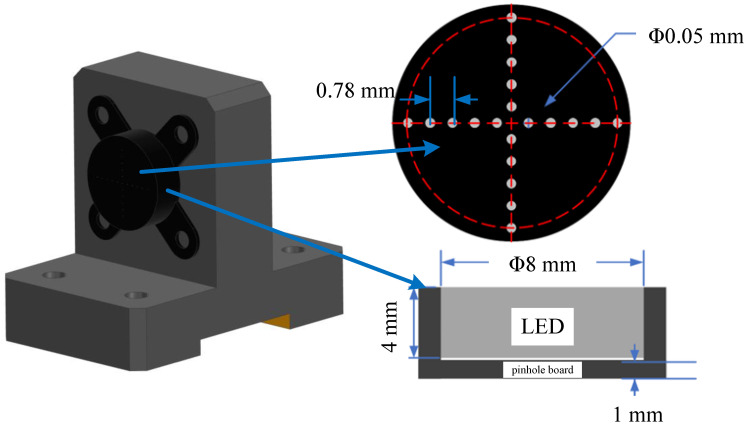
Design of target.

The targets are mounted on the support plates on both sides, with active targets employed to enhance measurement accuracy. After the camera captures images of the active targets, the specialized patterns on these targets, combined with corresponding image processing algorithms, enable high-precision calculation of the targets’ geometric positions. The active target design is illustrated in Figure 10. Each star-point target consists of an LED light source and a star-point target plate. The LED light source is cylindrical with dimensions of ϕ8×4 mm. The star-point target plate is manufactured from materials with varying transmittance. Each LED light source employs a constant-current drive with an adjustable current design, allowing LED current magnitude to be modified as required. Each LED light source incorporates four LED chips using Nichia NSSL146AT LEDs. The camera captures images of the active targets. The imaging signal-to-noise ratio reaches 30 dB, with each target consuming 0.8 W of power. The target support frame is made of carbon fiber, while the pinhole board is constructed from Invar. Material parameters are listed in Table 8.

### 3.7. Measurement and Wavefront Fitting Algorithm Design

#### 3.7.1. Target Center Calculation

**Figure 11:**
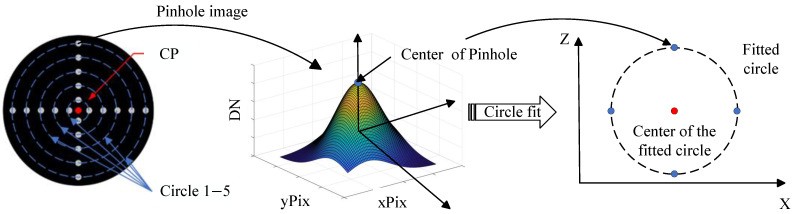
The process of calculating the center of the target.

To enhance the measurement accuracy of the target’s center point (CP), multiple small holes (pinhole apertures) were incorporated into a single star-point target, as shown in Figure 11.

In Figure 11, CP represents the center point of the target. The centers of fitted circles 1 through 5 are calculated, and the average of these five center positions is taken. Multiple fittings are performed to enhance the computational precision of the CP coordinates. The specific steps are as follows:

1. Extract the grayscale values of the pinhole image to form the point spread function (PSF). Fit the PSF to a two-dimensional Gaussian surface using the least squares method as Equation (1). Determine the position of the Gaussian surface’s peak, which corresponds to the coordinates of the pinhole center point within the image coordinate system.
  (1) $$L\left(A,xPix_{0},yPix_{0},\sigma ,c\right)=\sum_{i}^{n}{\left[DN_{i}-\left(Ae^{-\frac{{\left(xPix_{i}-xPix_{0}\right)}^{2}+{\left(yPix_{i}-yPix_{0}\right)}^{2}}{2\sigma^{2}}}+c\right)\right]}^{2}$$
  In the equation, A represents the amplitude of the height surface, σ denotes the standard deviation of the Gaussian surface, c signifies the offset of the Gaussian surface, and $\left(xPix_{0},yPix_{0}\right)$ indicates the coordinates of the pinhole center point in the image coordinate system; $\left(xPix_{i},yPix_{i}\right)$ and $DN_{i}$ denote the pixel position and grayscale value of the pinhole image, respectively:
2. Calculate the center coordinates of the four pinhole images on each ring using the same procedure, and fit the center positions as shown in the upper left image;
3. Repeat Step 1 to determine the positions of the four blue points shown in the upper right image;
4. Fit the center positions based on the four blue points;
5. After fitting and calculating the positions of the five centers, take the average as the CP position in the image coordinate system.

#### 3.7.2. Common Reference Transformation of Target

**Figure 12:**
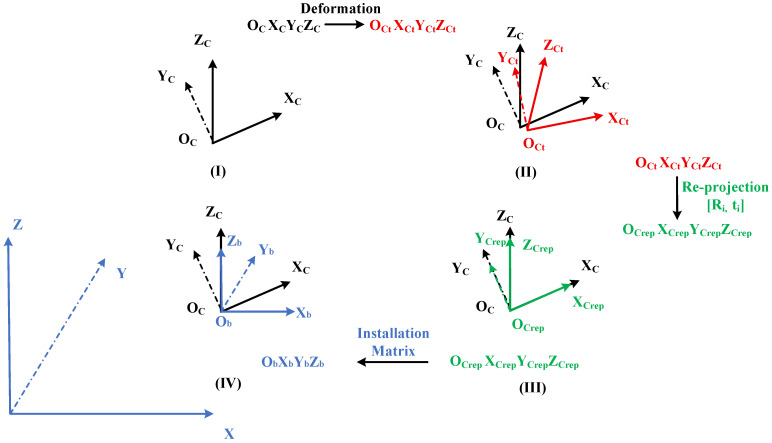
The process of common reference transformation of camera. (**I**) Camera coordinate system. (**II**) Camera coordinate system error caused by deformation. (**III**) Camera coordinate system corrected by reprojection. (**IV**) Transformation to the common reference frame.

After the four cameras measure the target’s coordinates, they each obtain four position matrices $H_{i}$ within their respective camera coordinate systems. To fit the overall surface profile of the folding mechanism, these four position matrices must be mapped to a common reference coordinate system via matrix transformation. The four camera coordinate systems and the reference coordinate system are shown in Figure 12I; due to the deformation of the folding mechanism, the four cameras undergo translation and rotation, causing corresponding shifts in their respective camera coordinate systems, as illustrated in Panel (II); using the reprojection algorithm, the external parameter matrices [$R_{i}$, $t_{i}$] of the four cameras relative to the reference target are calculated [23,24]. Based on these external parameter matrices, the position matrices measured by the four cameras undergo translation and projection to obtain the reprojected position matrices, as shown in Panel (III); the reprojected position matrices are then transformed into the reference coordinate system of the folding mechanism via the position mounting matrix, yielding the target’s position matrix $H_{i}^{xyz}$ in the common reference frame, as shown in Panel (IV).

**Figure 13:**
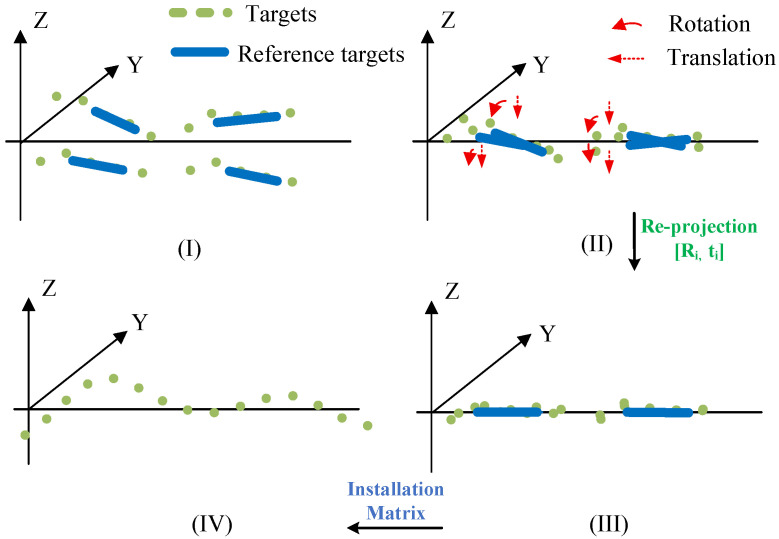
The process of common reference transformation of target: (**I**) Target position in the camera coordinate system; (**II**) Perform a reprojection transformation of the target position using the extrinsic parameter matrices of the four cameras relative to the reference target; (**III**) Target position after reprojection; (**IV**) Transform the target position to the reference coordinate system of the folding mechanism via the installation matrix.

The preceding content describes the transformation process of camera coordinates during the target co-reference procedure. To clarify the co-reference process further, Figure 13 illustrates the transformation process of target coordinates. All matrices and operations depicted in Figure 12 correspond to those in the preceding diagram.

After co-referencing, the target’s position matrix $H^{xyz}$ is obtained. The position matrix $H^{xyz}$ has dimensions 96 × 3, with each row representing the (X, Y, Z) coordinates of the target’s center.

#### 3.7.3. Wavefront Fitting

The key to Wavefront fitting for a segmented reflector lies in selecting appropriate polynomials that can accurately approximate the actual characteristics of surface deformation. Additionally, high numerical stability is required during polynomial fitting to enhance the computational accuracy of the fitting algorithm [25]. The process of describing surface deformation using polynomials can be expressed by Equation (2).

(2) $$\begin{array}{c} H^{xyz}=CB\\ C={\left[c_{1}c_{2}c_{3},\ldots ,c_{n}\right]}^{T}\\ B=\{b_{1}b_{2}b_{3},\ldots ,b_{n}\} \end{array}$$

In the equation, B denotes the polynomial basis, and C represents the polynomial coefficients. The basis depends solely on the polynomial type. After the target position undergoes a common reference transformation, $H^{xyz}$ also becomes a known quantity. Therefore, by solving for the polynomial coefficients C, surface deformation fitting can be completed.

**Figure 14:**
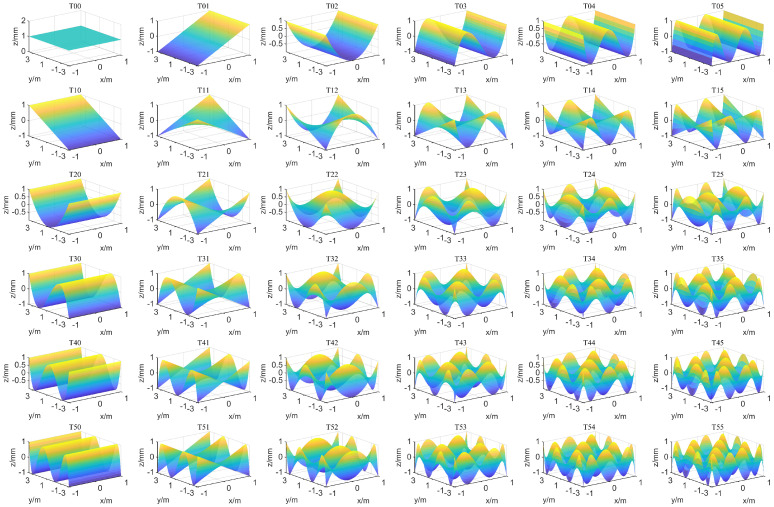
The graph of the Chebyshev function.

In the field of optics, Zernike polynomials are commonly used to describe optical surface deformations. At their core, they approximate surface deformations using orthogonal bases within the unit circle in polar coordinates. Each basis of the Zernike polynomials corresponds to a specific Seidel aberration. Due to the orthogonality of the bases, different types of aberrations can be separated. Therefore, Zernike polynomials are widely applied in the design, fabrication, and calibration of circularly symmetric optical elements [26]. However, within rectangular domains, the Zernike bases lose orthogonality. Coupling is most severe at the four corners of the rectangle, and the non-orthogonality between bases leads to ill-posed equations when solving for polynomial coefficients, compromising solution accuracy. Chebyshev polynomials maintain orthogonality within rectangular domains, with each basis corresponding to a distinct surface shape [27]. The basis for the 25th-order Chebyshev polynomial is illustrated in Figure 14.

The Table 9 above provides the expressions for the first 15 Chebyshev polynomials. It can be seen that the polynomial expressions correlate well with the aberrations under unidirectional light focal length, and the orthogonally oriented aberrations can be effectively separated.

**Figure 15:**
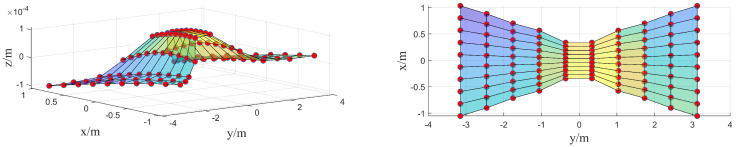
Non-uniform grid distribution, 3D view on the left, 2D view on the right.

The Chebyshev polynomial’s orthogonal basis forms a uniform grid. However, due to camera field-of-view limitations, targets are not distributed uniformly across this grid. Interpolation is therefore required to map the target distribution onto a uniform grid. First, the nearest-neighbor interpolation algorithm is used to compute the sparse surface equation, as illustrated in Figure 15.

**Figure 16:**
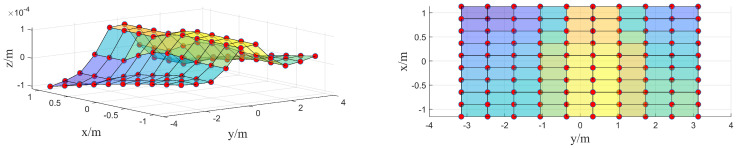
Mapped uniform grid distribution, 3D view on the left, 2D view on the right.

Resampling the target positions based on surface equations maps the target distribution onto a uniform grid distribution, as shown in Figure 16.

**Figure 17:**
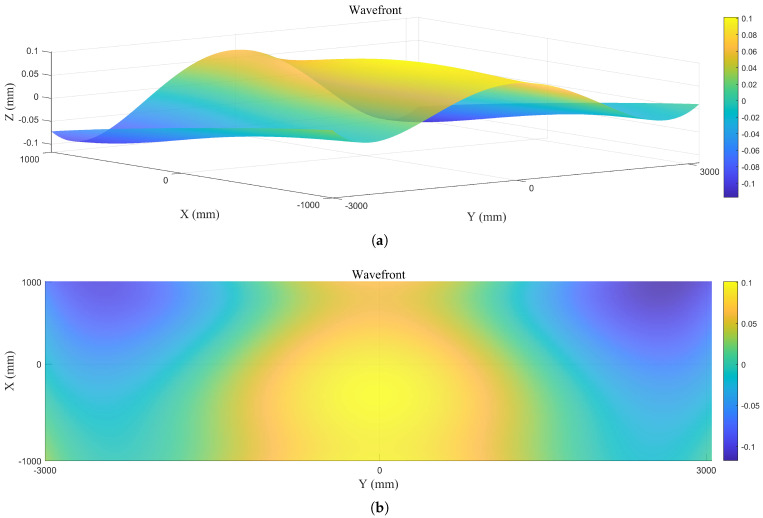
Wavefront after Chebyshev polynomial fitting: (**a**) 3D View. (**b**) 2D View.

The coefficients of Chebyshev polynomials were calculated using the least squares method to obtain the wavefront after deformation of the folding mechanism, the 3D and 2D views are shown in Figure 17a and Figure 17b, respectively.

## 4. Measurement Error Analysis

The measurement accuracy of target position is primarily influenced by focal length, distortion, target mounting errors, thermal deformation of the target, image signal-to-noise ratio, and deployment errors of the folding mechanism. These factors are categorized into random errors and calibratable errors. Random errors affect the measurement accuracy of the target position, while calibratable errors do not impact measurement accuracy after calibration.

### 4.1. Focus Error

During actual manufacturing, factors such as curvature errors in lens processing and alignment errors cause discrepancies between the lens’s actual focal length and its theoretical value. This discrepancy can be calibrated on the ground using a total station and a striped target [28]. After calibration, the focal length error does not affect measurement accuracy.

### 4.2. Distortion

**Figure 18:**
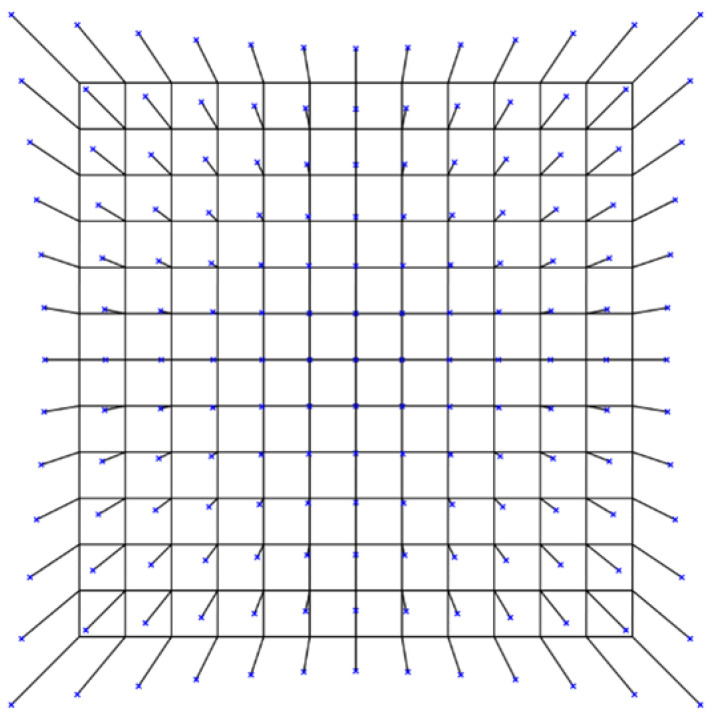
Optical distortion: The black grid represents the theoretical image, while the blue dots indicate the distorted image (100× magnification).

Due to optical distortion, the position of the target at the image edge deviates from its actual location, resulting in measurement errors in the target’s position, as shown in Figure 18.

Optical distortion can be calibrated using a standard calibration plate, and the resulting calibration data can be applied to correct image distortion. After correction, image distortion does not affect measurement accuracy.

### 4.3. Noise of Image

**Figure 19:**
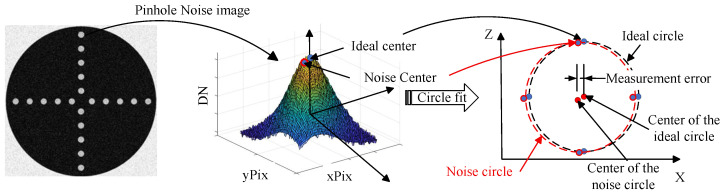
The process of calculating the center of the target, because of noise image.

Image noise is one of the primary factors affecting the accuracy of center localization. As illustrated in Figure 19. Under ideal conditions, the target center point corresponds to the center of an ideal Gaussian curve. However, under the influence of image noise, the center of the curve shifts, leading to errors in center calculations.

Image signal-to-noise ratio is a key factor affecting the accuracy of target center position estimation. Gaussian random noise was added to target images to achieve signal-to-noise ratios ranging from 20 to 40 dB. Target center coordinates were calculated, with 5000 simulations conducted for each SNR level. The relationship between signal-to-noise ratio and target center calculation error is shown in Table 10.

Results indicate that as the image signal-to-noise ratio increases, the calculation error for the target point’s center decreases. When the image signal-to-noise ratio reaches 30 dB, the horizontal and vertical calculation errors for the center are $e_{p}=\pm 0.1$ px. The measurement error caused by the center calculation error is defined as Equation (3). Similarly, $e_{y}$.

(3) $$e_{z}=\frac{vL}{f}dy-\frac{(v+e_{p})L}{f}dy=-\frac{ve_{p}}{f}dy$$

In the equation, $e_{z}$ represents the measurement error, while *v*, *f*, dy, and *L* denote the image height of the target, the focal length of the lens, the pixel size of the detector, and the measured object distance, respectively.

### 4.4. Folding Mechanism Deployment Error

**Figure 20:**
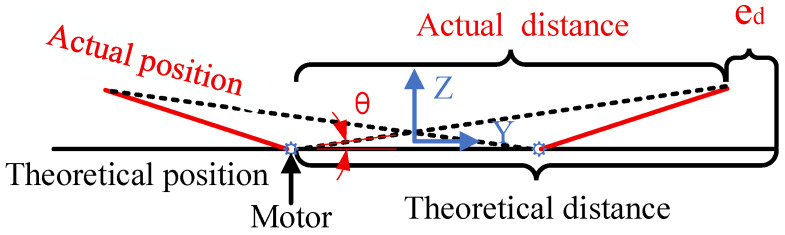
Folding mechanism deployment error.

After the folding mechanism enters the track and unfolds, due to errors in the unfolding mechanism, the unfolded angle of the folding mechanism deviates from the theoretical value. This angular error affects the object distance of the target, leading to measurement errors. The unfolding errors of the folding mechanism around the X-axis are shown in Figure 20.

In Figure 20, the black line indicates the theoretical position of the folding mechanism after deployment, while the red line shows its actual position. θ represents the deployment angle error, and $e_{d}$ denotes the target distance error caused by the folding mechanism’s deployment angle error. Maximum RX tilt error: 0.5°, resulting in a distance error at 4 m: 4−4×cos(0.5°)=0.152 mm. Similarly, the maximum RZ tilt error is 0.5°, resulting in a distance error at 4 m of 0.152 mm. The RZ tilt error and RX tilt error are synthesized as $e_{slant}=\pm \sqrt{0.152^{2}+0.152^{2}}=\pm 0.251$ mm. Measurement errors caused by tilt are defined as Equation (4). Similarly, $e_{x}$.

(4) $$e_{z}=\frac{vL}{f}dy-\frac{v(L+e_{slant})}{f}dy=-\frac{ve_{slant}}{f}dy$$

### 4.5. Deformation Error of Folding Mechanism

After the folding mechanism enters the orbit, thermal deformation of the mechanism causes errors in the object distance of the target. According to thermal simulation results of the folding mechanism, the maximum thermal deformation of the folding mechanism along the Y-axis and X-axis falls within ±0.8 mm and ±0.3 mm, respectively. The Y-axis represents the line of sight of the measurement camera, affecting the measured object distance, while the X-axis deformation does not impact the measured object distance. According to the following equation, the target object distance error caused by the thermal deformation of the folding mechanism is $e_{thermal}=\pm 0.8$ mm. Measurement errors caused by thermal deformation are defined as Equation (5). Similarly, $e_{x}$.

(5) $$e_{z}=\frac{vL}{f}dy-\frac{v(L+e_{thermal})}{f}dy=-\frac{ve_{thermal}}{f}dy$$

### 4.6. Target Installation Error

The target is mounted on a folding mechanism. Due to machining errors in the mounting holes, the actual installation position of the target deviates from its theoretical position. This causes an error in the measured object distance, leading to measurement errors in the target’s Z-axis position. The target mounting error can be calibrated using a total station and a laser interferometer. After calibration, the target mounting error does not affect measurement accuracy.

### 4.7. Target Thermal Deformation Error

After the target enters orbit, thermal deformation along the Z-axis occurs due to temperature effects, directly causing measurement errors. The target bracket has a height of 50 mm and is made of carbon fiber with a thermal expansion coefficient of $10^{-6}$/K. The target’s thermal environment is ±10 °C. The thermal deformation along the Z-axis is calculated as $e_{z}=\pm 50\times 10^{-6}\times 10=\pm 0.005$ mm.

### 4.8. Camera Installation Error

The camera is mounted on a folding mechanism. Due to manufacturing tolerances in the mounting holes and camera support structure, the actual position of the camera’s optical axis deviates from its theoretical position. This results in camera mounting error, which introduces measurement error in the target’s Z-axis position. Camera mounting error can be calibrated using a total station and reference prism. After calibration, the camera mounting error does not affect measurement accuracy.

### 4.9. Camera Thermal Deformation Error

After the measurement camera entered orbit, temperature variations caused changes in the focal length of its lens. Under the combined effects of the camera’s thermal control system and the orbital thermal environment, the camera temperature was maintained at 20±4 °C. The lens, constructed from titanium alloy with a thermal expansion coefficient of $7.9\times 10^{-6}$/K, exhibited focal length changes at different temperatures as shown in Table 11.

Measurement errors caused by focal length variation are defined as Equation (6). Similarly, $e_{x}$.

(6) $$e_{z}=\frac{vL}{f}dy-\frac{v(L)}{f+f_{e}}dy$$

Among these, $f_{e}$ represents the focal length error caused by temperature variation.

**Figure 21:**
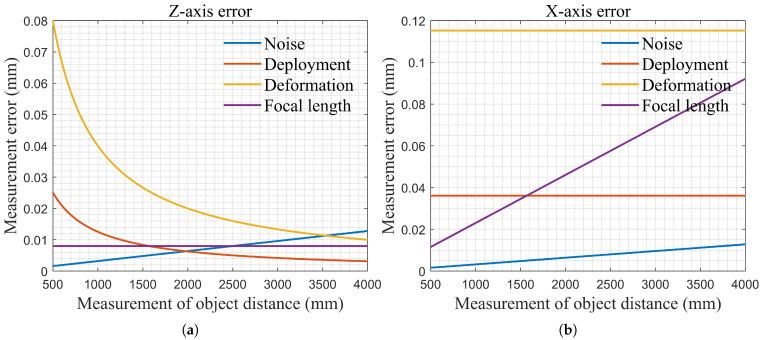
Target position measurement error: (**a**) Z-axis, (**b**) X-axis.

The measurement errors for the Z-axis and X-axis positions of the target at different object distances are analyzed separately. The variation curves of measurement errors with respect to object distance are shown in Figure 21.

The inputs for each error term are shown in Table 12. The measurement errors for the target’s Z and X positions are obtained by synthesizing the errors according to Equation (7).

(7) $$\begin{array}{c} e_{ztotal}=\sqrt{\sum (e_{z}^{i}{)}^{2}}\\ e_{xtotal}=\sqrt{\sum (e_{x}^{i}{)}^{2}} \end{array}$$

## 5. Experimental and Simulation Analysis

### 5.1. Measurement Camera Imaging Experimental

**Figure 22:**
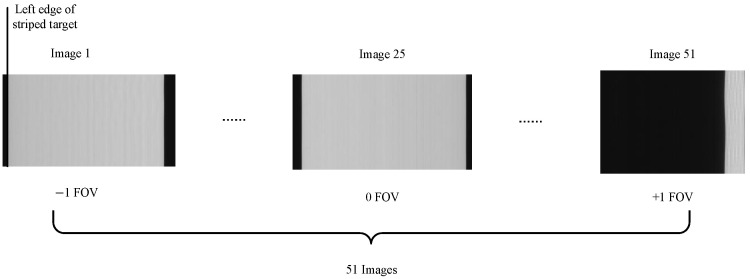
Calibration of the camera’s focal length.

To validate the focal length and imaging performance of the optical measurement camera designed in this paper, focal length calibration and imaging tests were conducted. First, the measurement lens was manufactured and assembled according to the camera design specifications, with the GMAX3265 selected as the imaging electronics component. A prototype target was machined based on its dimensional parameters. Focal length calibration tests were performed using a collimator and precision turntable, followed by integrated camera testing and target imaging experiments. The lens focal length was calibrated using a stripe target, collimator, and precision turntable. The stripe target was positioned at the collimator’s focal plane to ensure parallel light incidence into the measurement camera. The precision turntable was controlled to rotate the camera in random increments, moving the grating target from the far left to the far right of the image. Fifty-one images were captured as shown in Figure 22, with the turntable’s rotation angle recorded.

The camera focal length was calculated using the Equation (8), yielding a final value of 101.356 mm.

(8) $$\frac{1}{N-1}\sum_{i=1}^{N}\frac{(I_{i+1}-I_{i})d}{R_{i+1}-R_{i}}$$

**Figure 23:**
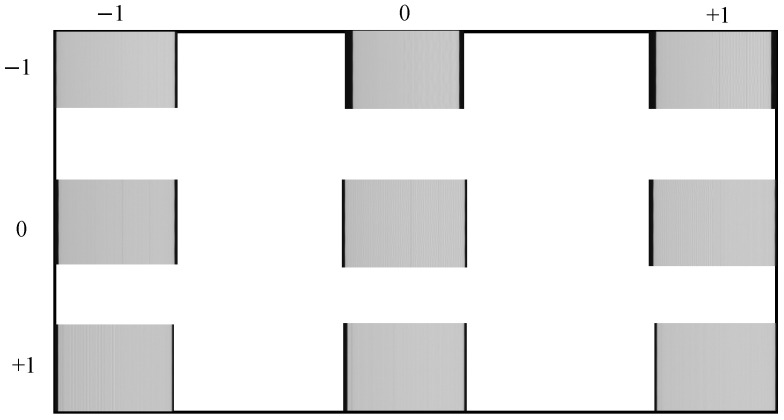
Camera MTF test.

In the Equation (8), *N* represents the number of captured images, *d* denotes the pixel size, *R* is the rotation angle of the turntable, and *I* indicates the position of the leftmost pixel in the fringe pattern image. The MTF of the camera’s central and edge FOV was tested using a fringe pattern target and a parallel light tube. A precision turntable rotated the camera, positioning the fringe pattern target within each of the camera’s nine FOVs. As shown in Figure 23.

The MTF for the nine FOVs was calculated using the Equation (9), with results presented in Table 13,

(9) $$\frac{DN_{max}-DN_{min}}{DN_{max}+DN_{min}}$$

In the Equation (9), $DN_{max}$ and $DN_{min}$ represent the maximum and minimum gray values of adjacent pixels, respectively.

The GMAX3265’s target plane dimensions are 29.4912×22.9376 mm. Based on focal length calibration results, the camera’s measured FOV is 16°×12°. The measurement range of the optical system primarily depends on the closest target (500 mm). To ensure effective measurement, the Z-axis positional variation in the target caused by folding mechanism deformation must not exceed the FOV. At 500 mm, the camera’s Z-axis FOV width is 105 mm, and the target diameter is 8 mm. Therefore, the permissible Z-axis position variation for the target is ±48.5 mm, significantly exceeding the design specification. Similarly, at 500 mm, the measurement camera’s X-axis FOV width is 140.5 mm. Targets are positioned within the camera’s ±7° X-axis FOV, spanning a width of 122.784 mm. Considering the target diameter, the measurement camera permits a ±4.85 mm Z-axis position variation for the target, meeting the design specifications.

### 5.2. Wavefront Fitting Deviation Analysis

**Figure 24:**
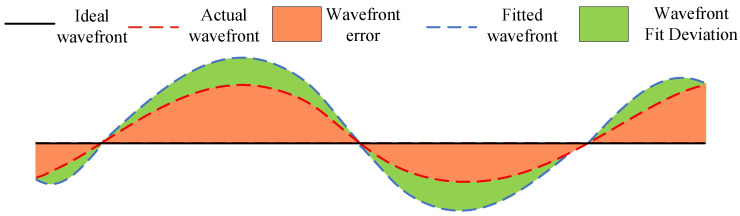
Wavefront aberration and wavefront fitting deviation.

After deployment of the folding mechanism and sub-mirror into orbit, the temperature deviation of the folding mechanism relative to ground testing conditions will remain within ±10 °C due to orbital thermal effects. The position of the sub-mirror will shift with temperature variations, and this positional error will induce wavefront errors in the assembled mirror, as illustrated in Figure 24.

To validate the accuracy of the proposed measurement system and wavefront fitting algorithm for fitting the wavefront of the tilting mirror under actual operating conditions, finite element analysis was employed. Under conditions of ±0.5° deployment error and ±10 °C temperature variation, the deformation of the secondary mirror was analyzed. The wavefront was then fitted, and the wavefront fitting deviation was calculated. The definition of the fitting deviation is illustrated in Figure 24.

The fitting accuracy is evaluated by assessing the RMS and PV values of the fitting deviation. These are defined as Equation (10):

(10) $$\begin{array}{c} PV=Max(Dev.)-Min(Dev.)\\ RMS=\sqrt{\frac{\sum_{i=1}^{N}\left(Dev._{i}-\bar{Dev.}\right)}{N}} \end{array}$$

The fitting deviation of the mirror surface is primarily influenced by measurement errors in target position and common-reference transformation errors. The deviation analysis process is as follows:

1. Establish a finite element model of the folding mechanism to calculate its deformation under different temperature conditions. The global coordinate system of the finite model serves as the reference coordinate system for the folding mechanism;
2. Set measurement points at the camera installation location within the finite element model. Output the positions of nodes at the camera installation location in the reference coordinate system under various temperature conditions;
3. Set measurement points at the target installation position within the finite element model and output the node positions at the target installation location in the reference coordinate system under different temperature conditions;
4. Establish the coordinate systems for each of the four cameras based on the deformed positions of the nodes at the camera installation locations;
5. Map the target positions in the reference coordinate system to the coordinate systems of the four cameras;
6. Superimpose the target’s X- and Z-axis position error analysis results from Table 12 onto the target position within the camera coordinate systems. Since the target’s Y-axis position is unmeasurable for the measurement camera in this study, directly superimpose the thermal deformation of the target’s Y-axis onto its position within the camera coordinate systems;
7. Perform a common reference transformation on the target positions in the four camera coordinate systems to obtain the target position in the reference coordinate system;
8. Fit the surface deformation of the folding mechanism using Chebyshev and Zernike polynomials, respectively;
9. Compare the finite element analysis results with the fitted deformation to obtain the fitting deviation;
10. For finite element analysis results under different temperature conditions, repeat steps 6–9 to perform 10,000 Monte Carlo analyses;

The average RMS and PV fitting deviations of Chebyshev and Zernike polynomials under various operating conditions are shown in Table 14; under 20 simulated operating conditions, the maximum fitting deviation using Chebyshev polynomials was 0.397 mm (PV) and 0.073 mm (RMS), while the maximum fitting deviation using Zernike polynomials was 0.446 mm (PV) and 0.089 mm (RMS). Chebyshev polynomials demonstrated superior fitting accuracy for the unfolding error of the folding mechanism and the wavefront error of the assembled mirror caused by thermal deformation.

The Monte Carlo analysis in Table 14 uses the maximum measurement error of the measuring camera as the input value, which is a more conservative approach. To make the error analysis results more representative of real-world conditions, the measurement error of the optical camera was input based on the object distance. The resulting wavefront fitting deviation is shown in Table 15; under 20 simulated operating conditions, using the optical measurement error corresponding to the object distance as input, the maximum fitting deviation of Chebyshev polynomials was 0.263 mm (PV) and 0.049 mm (RMS), while that of Zernike polynomials was 0.343 mm (PV) and 0.067 mm (RMS). Both values are smaller than the analysis results in Table 14.

## 6. Discussion

Compared to existing wavefront measurement methods for tiled mirrors, the measurement system proposed in this paper offers distinct advantages in core performance metrics and application scenarios. These advantages are primarily manifested in its capability to cover a wide error range during the coarse calibration phase and its exceptional environmental adaptability. The common-phase calibration of tiled mirrors is typically divided into two stages: coarse and fine adjustment. Existing mainstream wavefront measurement technologies (such as JWST, KECK, TMT, and DFS systems) primarily focus on final high-precision imaging quality correction, with relatively limited measurement ranges.

As shown in Table 16 below, while the Shack–Hartmann sensors used by KECK and TMT achieve extremely high precision (50 nm and 30 nm RMS, respectively), their measurement range is limited to ±30 μm. This means that during initial sub-mirror installation or after significant environmental changes, if positional deviations exceed tens of micrometers, these high-precision sensors become ineffective due to exceeding their dynamic range. JWST extends its measurement range to ±500 μm through dispersive Shack–Hartmann and dispersive fringe sensors, while ELT achieves a 400 μm monitoring range using inductive edge sensors. However, these techniques often still rely on complex iterative search algorithms to detect signals when confronting millimeter-level initial position errors.

In contrast, the measurement system proposed in this paper boasts a measurement range of up to ±4 mm—eight times greater than JWST’s and two orders of magnitude larger than KECK/TMT’s. Although its 0.1 mm (100 μm) RMS measurement accuracy is numerically lower than optical interferometry, this “wide-range, medium-accuracy” characteristic is precisely what the system urgently requires during the coarse calibration phase when secondary mirror position errors are substantial. It enables rapid, one-time convergence of large-scale positional errors, adjusting the secondary mirror into the detectable range of high-precision sensors (such as Shack–Hartmann) and bridging the gap between initial installation and fine calibration.

## 7. Conclusions

This paper analyzes the static and dynamic characteristics of the measurement camera through simulation, evaluating the surface accuracy of the camera lens under gravitational and thermal rise conditions. A prototype was manufactured, and an experimental platform was constructed to conduct imaging tests. Finite element simulation was employed to analyze the fitting accuracy of the mirror wavefront fitting algorithm for the stitching process. The following conclusions can be drawn from the simulation and experimental results:

1. Under conditions of gravity and a 4 °C temperature increase, the RMS surface profile of the mirror aperture achieves better than 1/80λ. The measurement camera structure designed in this paper operates at a fundamental frequency of 464.1 Hz, meeting the requirements for its use in space environments;
2. Based on imaging models and error theory, this study analyzes the impact of signal-to-noise ratio on target center calculation errors, along with the impact of object-to-camera distance errors caused by folding mechanism deployment and thermal deformation, and the focal length error of the measurement camera on the final target position measurement accuracy. The error analysis results indicate measurement accuracies of ±0.0853 mm in the Z-direction and ±0.1525 mm in the X-direction, laying the foundation for subsequent wavefront fitting accuracy analysis;
3. A prototype measurement camera was constructed and underwent focal length calibration tests and MTF testing using a stripe target, parallel light tube, and precision turntable. The camera’s focal length was determined to be 101.356 mm, with MTF values exceeding 0.11 across all nine FOVs. Based on the GMAX3265 calibration results for dimensions and focal length, the camera’s FOV was determined to be 16° × 12°. Considering the distribution pattern of the test targets, the measurement system’s range was established as ±4.85 mm. These results confirm that the measurement camera’s focal length, MTF, and measurement range all meet the design specifications;
4. A target common-reference transformation algorithm was designed. Considering the transformation of the cameras’ own mounting positions, it uses a common target as the reference to transform the target position measurement results from the four cameras in their respective coordinate systems to a single common reference coordinate system. Based on the rectangular aperture of the stitching mirror, Chebyshev polynomials were selected for wavefront fitting. Finite element analysis validated the effectiveness of the target common-reference transformation algorithm and wavefront fitting algorithm using RMS and PV fitting deviations as evaluation criteria, with comparisons against Zernike polynomials. Under various operating conditions, the Chebyshev polynomial achieved maximum wavefront fitting deviations of 0.397 mm (PV) and 0.073 mm (RMS), meeting the wavefront measurement accuracy requirements for the coarse calibration stage. Meet the accuracy requirements for wavefront measurement during the coarse alignment phase of the mirror assembly (RMS < 0.1 mm, PV < 0.45 mm). The Chebyshev polynomial demonstrated superior fitting accuracy compared to the Zernike polynomial, proving more suitable for wavefront fitting in rectangular apertures.

## Figures and Tables

**Table 1:** PV value of wavefront for segmented mirrors under thermal deformation and deployment error effects (mm).

Temperature	10	11	12	13	14	15	16	17	18	19
PV	2.998	2.954	2.822	2.791	2.359	2.314	2.288	2.094	2.069	2.034
Temperature	21	22	23	24	25	26	27	28	29	30
PV	3.404	3.426	3.431	3.437	3.556	3.627	3.658	3.739	3.775	3.785

**Table 2:** Key parameters of the measurement system.

Item	Performance
Number of Targets	96
Number of Cameras	4
Wavefront measurement range	±4 mm
Wavefront measurement accuracy	Deviation RMS < 0.1 mm, PV < 0.45 mm

**Table 3:** Materials.

Materials	Modulus of Elasticity (MPa)	Poisson Ratio	Density $\rho ({g/\mathrm{cm}}^{3})$	Linear Coefficient of Thermal Expansion (*K*^−1^)	Application Components
TC4	109,000	0.29	4.4	$2.5\times 10^{-6}$	Supporting frame
T700	60,000	0.28	1.6	$1.0\times 10^{-6}$	Lens hood

**Table 4:** Composition of mass.

Components	Mass (kg)
Structural components	0.70
Optical components	0.085
Detector assembly	0.4
Cables, screws, adhesives, etc.	0.1
Total mass	1.235

**Table 5:** Optomechanical integration analysis results.

Conditions	PV (nm)	RMS (nm)
Gravity	0.044	0.007
Thermal	22.557	3.244

**Table 6:** Key Performance Characteristics of Imaging Electronics.

Item	Performance
CMOS pixel size	3.2 μm
Image dimensions	9216 × 7168
Quantitative digit	12 bit
Power consumption	5 w

**Table 7:** Camera active thermal control with zone-specific heating targets.

Heating Zone Position	Temperature Control Target (°C)
Backend Lens Element	21
Front Lens Element	20
Electronics Assembly	19

**Table 8:** Material of target.

Materials	Modulus of Elasticity (MPa)	Poisson Ratio	Density ρ (g/cm^3^)	Linear Coefficient of Thermal Expansion (K^−1^)	Application Components
4J32	140,000	0.29	8.1	$0.5\times 10^{-6}$	Pinhole board
T700	60,000	0.28	1.6	$1.0\times 10^{-6}$	Base of target

**Table 9:** Chebyshev polynomials and aberration correspondence.

$T_{n}\left(x\right)\times T_{m}\left(y\right)$	Chebyshev Polynomial	Corresponding Aberration
00	1	Piston
10	*x*	X-Slant
01	*y*	Y-Slant
20	$2x^{2}-1$	X-defocus
11	xy	
02	$2y^{2}-1$	Y-defocus
30	$4x^{3}-3x$	X-coma
21	$(2x^{2}-1)y$	
12	$(2y^{2}-1)x$	
03	$4y^{3}-3y$	Y- coma
40	$8x^{4}-8x^{2}+1$	X-Spherical aberration
31	$(4x^{3}-3x)y$	
22	$\left(2x^{2}-1\right)\left(2y^{2}-1\right)$	
13	$(4y^{3}-3y)x$	
04	$8y^{4}-8y^{2}+1$	Y-Spherical aberration

**Table 10:** The relationship between SNR and target center calculation error.

SNR (dB)	Center Error (Pixel)
20	±0.2903
22	±0.2305
24	±0.1829
26	±0.1453
28	±0.1155
30	±0.0918
32	±0.0728
34	±0.0578
36	±0.0459
38	±0.0366
40	±0.0290

**Table 11:** Changes in lens focal length at different temperatures.

Temperatures (°C)	Changes in Focal Length (mm)
24	+0.016
22.5	+0.010
17.4	−0.010
16	−0.015

**Table 12:** Measurement error analysis results.

Item	Error Value	Maximum Measurement Error Z (mm)	Maximum Measurement Error X (mm)	Error Type
Focus error	±0.1 mm	0	0	Calibratable
Distortion	±0.5 px	0	0	Calibratable
Noise of image	±0.1 px	±0.0128	±0.0128	Random
Deployment error of folding mechanism	±0.251 mm	±0.0251	±0.0361	Random
Deformation error of folding mechanism	±0.8 mm	±0.08	±0.1152	Random
Target installation error	$\pm 2^{\prime }$	0	0	Calibratable
Target deformation error	±0.005 mm	±0.005	±0.005	Random
Camera installation error	$\pm 4^{\prime }$	0	0	Calibratable
Camera thermal deformation error	±0.016 mm	±0.008	±0.0921	Random
Total		±0.0853	±0.1525	

**Table 13:** MTF Test Results for the Measuring Camera.

FOV	−1	0	+1
−1	0.118	0.126	0.114
0	0.128	0.131	0.123
+1	0.115	0.122	0.112

**Table 14:** Monte Carlo analysis results for wavefront fitting deviation. (The error input is independent of the distance to the measured object).

Temperature	Average of RMS (mm)		Average of PV (mm)		Maximum of RMS (mm)		Maximum of PV (mm)	
°C	Chebyshev	Zernike	Chebyshev	Zernike	Chebyshev	Zernike	Chebyshev	Zernike
1	0.044	0.061	0.250	0.324	0.069	0.084	0.364	0.437
2	0.043	0.061	0.243	0.317	0.068	0.084	0.348	0.427
3	0.042	0.059	0.239	0.307	0.072	0.083	0.360	0.421
4	0.042	0.059	0.240	0.305	0.073	0.089	0.353	0.443
5	0.044	0.060	0.251	0.308	0.072	0.086	0.361	0.423
6	0.046	0.060	0.258	0.307	0.073	0.085	0.377	0.427
7	0.047	0.060	0.259	0.308	0.070	0.079	0.379	0.429
8	0.047	0.061	0.260	0.314	0.073	0.088	0.380	0.427
9	0.046	0.060	0.255	0.316	0.072	0.083	0.397	0.436
10	0.045	0.061	0.253	0.324	0.073	0.086	0.367	0.446
11	0.031	0.044	0.174	0.234	0.050	0.073	0.256	0.401
12	0.030	0.048	0.171	0.259	0.051	0.074	0.264	0.423
13	0.030	0.043	0.168	0.232	0.050	0.070	0.277	0.371
14	0.030	0.041	0.170	0.217	0.053	0.067	0.254	0.388
15	0.030	0.039	0.172	0.205	0.054	0.068	0.263	0.354
16	0.031	0.036	0.177	0.182	0.050	0.059	0.271	0.351
17	0.032	0.036	0.180	0.174	0.056	0.065	0.294	0.302
18	0.031	0.038	0.178	0.191	0.052	0.064	0.295	0.324
19	0.032	0.035	0.183	0.173	0.055	0.061	0.284	0.289
20	0.032	0.038	0.179	0.192	0.053	0.067	0.284	0.331

Red text indicates the maximum value in each column.

**Table 15:** Monte Carlo analysis results for wavefront fitting deviation. (The error input is related to the distance to the measured object).

Temperature	Average of RMS (mm)		Average of PV (mm)		Maximum of RMS (mm)		Maximum of PV (mm)	
°C	Chebyshev	Zernike	Chebyshev	Zernike	Chebyshev	Zernike	Chebyshev	Zernike
10	0.035	0.057	0.195	0.312	0.045	0.066	0.248	0.334
11	0.034	0.056	0.189	0.302	0.042	0.064	0.234	0.333
12	0.033	0.055	0.185	0.292	0.043	0.064	0.240	0.320
13	0.033	0.055	0.187	0.293	0.042	0.064	0.236	0.330
14	0.036	0.056	0.198	0.296	0.045	0.066	0.246	0.325
15	0.039	0.056	0.204	0.297	0.048	0.064	0.253	0.325
16	0.040	0.056	0.207	0.299	0.047	0.064	0.263	0.330
17	0.039	0.056	0.211	0.303	0.049	0.067	0.256	0.333
18	0.038	0.056	0.205	0.306	0.046	0.065	0.250	0.337
19	0.037	0.058	0.202	0.311	0.046	0.066	0.246	0.343
21	0.016	0.039	0.093	0.208	0.026	0.048	0.139	0.246
22	0.015	0.043	0.087	0.236	0.023	0.053	0.133	0.283
23	0.014	0.037	0.083	0.204	0.023	0.046	0.124	0.248
24	0.014	0.035	0.082	0.188	0.023	0.043	0.127	0.225
25	0.014	0.033	0.084	0.177	0.025	0.043	0.137	0.226
26	0.017	0.029	0.096	0.151	0.029	0.038	0.143	0.180
27	0.019	0.028	0.106	0.146	0.029	0.037	0.147	0.173
28	0.018	0.032	0.103	0.167	0.028	0.043	0.146	0.194
29	0.019	0.028	0.106	0.147	0.031	0.035	0.149	0.178
30	0.018	0.031	0.103	0.163	0.027	0.042	0.142	0.194

Red text indicates the maximum value in each column.

**Table 16:** Performance of wavefront measurement systems.

System	Measurement Range	Measurement Accuracy	Sensor Type
Ref. [9]	±500 μm	256 nm RMS	Dispersed Hartmann Sensors and Dispersed Fringe Sensing
Ref. [14]	±30 μm	50 nm RMS	Shack–Hartmann
Ref. [15]	±30 μm	30 nm RMS	Shack–Hartmann
Ref. [12]	±100 μm	100 nm RMS	Dispersed Fringe Sensing
Ref. [11]	400 μm	1 nm, temperature drift is 1.32 nm/°C	Inductive Edge Sensors
This paper	±4 mm	0.1 mm RMS	Photogrammetry

## Data Availability

The data presented in this study are available in article.
